# *RB1* gene mutations in Argentine retinoblastoma patients. Implications for genetic counseling

**DOI:** 10.1371/journal.pone.0189736

**Published:** 2017-12-20

**Authors:** Diana Parma, Marcela Ferrer, Leonela Luce, Florencia Giliberto, Irene Szijan

**Affiliations:** 1 Cátedra de Genética, Facultad de Farmacia y Bioquimica, Universidad de Buenos Aires. Buenos Aires. Argentina; 2 Division de Neurocirugia, Hospital de Clinicas “Jose de San Martin”, Universidad de Buenos Aires. Buenos Aires. Argentina; Odense University Hospital, DENMARK

## Abstract

Retinoblastoma (RB) is an inherited childhood ocular cancer caused by mutations in the tumor suppressor *RB1* gene. Identification of *RB1* mutations is essential to assess the risk of developing retinoblastoma in the patients´ relatives. Retinoblastoma is a potentially curable cancer and an early diagnosis is critical for survival and eye preservation. Unilateral retinoblastoma is mostly non-heritable and results from two somatic mutations whereas bilateral retinoblastoma is heritable and results from one germline and one somatic mutation, both have high penetrance, 90%. The purpose of this study was to identify causative *RB1* mutations in RB patients with different clinical presentations. A comprehensive approach was used to study a cohort of 34 patients with unilateral, bilateral and trilateral retinoblastoma. Blood and tumor DNA was analyzed by sequencing and multiplex ligation-dependent probe amplification (MLPA) assay. Validation of an insertion mutation was performed by cloning the PCR product. Most of the patients in our cohort had unilateral RB, eight patients had bilateral RB and one patient had a trilateral tumor with ocular and suprasellar/sellar locations. Other tumors in addition to retinoblastoma were also found in the affected families. One patient had two syndromes, retinoblastoma and schwannomatosis, and another RB patient had a father with a retinoma. Five out of the 25 unilateral RB patients carried germinal mutations (20%), which were mostly missense mutations. The bilateral and trilateral patients carried splice-site, nonsense and frameshift mutations as well as a whole *RB1* gene deletion. Missense mutations were associated with mild phenotype: unilateral retinoblastoma, retinoma or no tumor. In this study we identified causative *RB1* mutations in most bilateral RB patients and in some unilateral RB patients, including five novel mutations. These data are crucial for genetic counseling and confirm the need to perform complete genetic screening for *RB1* mutations in both constitutional and tumor tissues.

## Introduction

Retinoblastoma (RB) is a malignant ocular childhood tumor originating from retinal cell progenitors and its incidence is approximately 1 case for every 15,000–28,000 live births [1]. Retinoblastoma develops as a result of inactivation of the tumor suppressor *RB1* gene, 40% of RBs are heritable tumors and 60% are non heritable tumors. In heritable RB the first *RB1* mutation is germline and the second mutation is somatic. In non-heritable RBs two somatic *RB1* mutations occur in the developing retina. Ten percent of heritable RBs are inherited and 30% arise “de novo”. In addition, 75–80% of heritable RBs are bilateral in which both eyes are affected and 15–25% are unilateral in which, only one eye is affected. Heritable RB can be diagnosed at approximately one year of age, whereas, non-heritable RB is always unilateral and develops at approximately two years of age or older [2,3].

Individuals with germline mutations are hereditarily predisposed to retinoblastoma, thus identification of the causative mutation is important to predict the risk for tumor development in patient´s relatives [4]. Given that RB is a potentially curable cancer early diagnosis is critical for survival and eye preservation in children who carry the *RB1* mutation [5].

The presence of an *RB1* germline mutation confers an increased risk for developing second primary tumors [6]. Midline intracranial primitive neuroectodermal tumors, such as pineal or suprasellar generally arise months to years after RB diagnosis [7]. Osteosarcomas and soft-tissue sarcomas usually arise during adolescence, whereas melanomas tend to occur in older patients [8]. The *RB1* mutation can also cause a rare benign retinoma tumor at a frequency of approximately 8.5% [9,10]. Retinoblastoma may also occur in association with other syndromes, such as Down syndrome (Trisomy 21) [11] or Schwannomatosis.

The human *RB1* gene was the first gene isolated with tumor suppressor activity and it is expressed in a wide variety of tissues [12,13]. The pRB protein product contains several functional domains, including highly conserved pocket domain that interacts with and inhibits E2F transcription factors, thereby preventing expression of genes required for the G1 to S phase transition [14,15,16,17]. Mutations in the *RB1* gene disrupt the structure and function of the pRB protein leading to deregulation of cell proliferation. The mutation spectrum ranges from large deletions to single-base substitutions and most are null mutations that result in the absence of pRB protein. The null mutations account for 90% of all of the *RB1* mutations and include nonsense, frameshift and splice-site mutations, whereas, missense, in-frame and promoter mutations are infrequent [18].

Retinoblastoma usually has a high penetrance, of 90%, because more than 90% of germline mutations lead to a lack of pRB protein and to development of tumors. However, some families display incomplete or low RB penetrance due to the type of mutation and environmental and lifestyle factors [19]. The *RB1* mutations associated with low penetrance include promoter mutations, missense mutations and in-frame deletions/insertions [19,20]. Furthermore, RB may present differentially among individuals with the same mutation which indicates variable expressivity [21]. It is essential to know the sequence variation that occur in *RB1* to understand the molecular mechanisms underlying the various manifestations of retinoblastoma, such as the different degrees of RB penetrance and expressivity.

Molecular genetic testing of RB patients identifies children with the heritable condition which includes ~50% of RB patients that can pass the mutation on to their children. Detection of germline mutations is particularly important in unilateral patients who are at risk of bilateralization [22]. In addition to detecting a predisposition for RB in pre-symptomatic siblings, it is important to detect non-carriers of RB mutations so that they can be excluded from clinical procedures that requires anesthesia.

This study is a continuation of our search for mutations in Argentine RB patients [11, 23, 24]. In this study we identified causative *RB1* mutations in patients with different clinical presentations. One of the patients presented with a rare trilateral retinoblastoma and another patient presented with an uncommon association of retinoblastoma and schwannomatosis. A comprehensive approach was used to identify the causative *RB1* mutations and to determine if they were heritable or non-heritable. These data were crucial to provide genetic counseling to the affected families and to obtain new insights into the cellular functions.

## Materials and methods

### Patients

Retinoblastoma patients were referred from children´s hospitals (JP Garrahan and R.Gutierrez) and other health care centers in Argentina. The RB diagnosis was established by current ophthalmologic/histological criteria. A total of thirty four retinoblastoma cases were studied, including twenty five unilateral (one of them associated with Schwannomatosis syndrome), seven bilateral, one trilateral patient and one familial RB. Informed consent for genetic analysis was signed by parents of the affected children according to the principles of the Declaration of Helsinki. The study was approved by ethics committee of “Hospital de Clinicas” of Buenos Aires, Argentina.

### DNA isolation and mutation analyses

DNA was obtained from peripheral blood leukocytes using the cetyltrimethylammonium bromide (CTAB) method as well as from frozen tumors by treatment with proteinase K, phenol/chloroform purification and ethanol precipitation.

Mutation screening was performed in blood DNA samples and in seven tumor DNA samples (obtained from patients with an available tumor biopsy, one bilateral and six unilateral). PCR-amplification and sequencing of the 27 exons, the promoter and the intronic flanking regions including an average of 50 bp (to encompase recognized splice sites) were performed using an ABI 3130XL analyzer [23]. All the mutations were confirmed by both directions sequencing from separate PCR-reactions, using as a reference for genomic alterations the *RB1* reference sequence L11910 (GeneBank accession number). The pathogenic effect of recurrent mutations and the novel mutations were confirmed from the specific database rb1-lsdb. Splice site alterations were predicted using the bioinformatic tool of “Human Splice Finder” (http://www.umd.be/HSF) and the prediction of functional effects of the novel missense and in-frame mutations was performed by “Mutation T@sting” (http://www.mutationtaster.org/) and Poly Phen 2 (http://genetics.bwh.harvard.edu/pph2/). Mutations were described according to the nomenclature of the Human Genome Variation Society (HGVS) and Den Dunnen and Antonarakis [25].

Multiplex Ligation-dependent Probe Amplification assay (MLPA) was performed using the Salsa MLPA kit P047-B1 RB1 (MRC Holland) according to the manufacturer´s protocol. The PCR amplicons were separated on ABI 3130XL analyzer and the results were analyzed using Coffalyser software. Loss of heterozygosity (LOH) was ascertained by the loss of one allele in the tumor DNA compared with the two heterozygous alleles in leukocytes´ DNA.

### Cloning of PCR products in pGEM-T vector

The vector contains thymidine residue (T) in the 3´end for its pairing with the (A) residue incorporated by Taq polymerase in PCR products. This vector also includes a multiple cloning site in the region encoding for α peptide of β galactosidase, inactivation of this gene by insertion of a PCR product allows the identification of the recombinant clones. Cloning was performed as described [23]. In brief, the PCR products were ligated to the vector pGEM-T and the mixture was transformed into DH5 α competent bacteria growing in a media with an inducer of β galactosidase (IPTG) and the chromogenic substrate 5-bromo-4chloro-3-indolyl-β galactoside (X-Gal). Recombinant vectors produced white colonies, while vectors without the insert originated blue colonies. The recombinant vector was extracted from white colonies and analyzed by digestion, electrophoresis and sequencing.

## Results

### Presentation, treatment and outcomes

Seventy four percent of patients studied had unilateral RB and the remainder had bilateral RB except one patient who had trilateral RB with a suprasellar/sellar neuroectodermal tumor in addition to bilateral RB. This patient was diagnosed at two months of age, enucleated and treated by chemotherapy, but despite the intensive treatments he died at two years of age. Another rare patient presented with two syndromes, unilateral RB and schwannomatosis. She underwent enucleation of the RB tumor at 18 months of age and later had two surgeries to remove the schwannoma tumors. She is currently an eighteen years old high school student. Upon analyses of the *RB1* and *SMARCB1* [26] genes in peripheral blood we did not find any germline mutation. As would be expected in unilateral RB and in schwannomatosis her mutations were somatic. One asymptomatic mutation carrier, father of retinoblastoma patient (#661), carried a rare benign tumor retinoma [9]. Twenty eight out of thirty four patients underwent enucleation. In contrast, five unilateral patients who were diagnosed before the age of one year were treated with chemotherapy and/or radiotherapy only. No reports were available for the remaining three patients. Most of the enucleated unilateral patients had no additional treatment, whereas the bilateral patients received chemotherapy in addition to enucleation (Table 1).

**Table 1:** Description of retinoblastoma patients with *RB1* gene mutations.

Patient ID	Phenotype	Age at diagnosis (months) Treatment	Tissue Analyzed	Mutation Description	Location exon/intron	Expected consequence	Recurrence / Heritability
**658**	Bilateral	9/Enucleation/ Chemotherapy	Tumor	1. g.2197G>A 2. LOH	Intron 1 IVS1+1G>A	Exon 1 skipped/Frameshift Stop codon p.E47X	Rare/Hereditary
			Blood	1. g.2197G>A			
**660**	Bilateral	24/Enucleation	Blood	1. g.2197G>A	Intron 1 IVS1+1G>A	Exon 1 skipped/Frameshift Stop codon p.E47X	Rare/Hereditary
				2. 156812-156813ins21bp	Exon 20:21bpins		Novel
**661**	Unilateral	22/Enucleation	Tumor	g.56913T>C (Heterozygous)	Exon 7	Missense: Leu>Pro Disruption of pRB structure	Novel/Hereditary
			Blood	g.56913T>C	Exon 7		
**Father**	Retinoma		Blood	g.56913T>C	Exon 7		
**3Relatives**	Asymptomatic		Blood	g.56913T>C	Exon 7		
**663**	Unilateral	9/Chemotherapy	Blood	1. g.61733/7delA	Exon 9:1bp del	Frameshift p.N290fs12X	Reported twice/ Hereditary
				2. g.156812-156813ins	Exon 20:21bp ins	In-frame (Mosaic)	Novel
**665**	Unilateral	34/Enucleation	Blood	g.2118C>T	Exon 1	Missense Pro>Leu Disruption of pRB structure	Reported twice/ Hereditary
**Mother**	Asymptomatic		Blood	g.2118C>T	Exon 1	Idem	
**666**	Trilateral/ Died at 2years	2/Enucleation/ Chemotherapy	Blood	g.56880delT	Exon7:1bpdel	p.L212fsX2	Reported once/ Hereditary
**668**	Unilateral	4years/Enucleation	Tumor	1. g.56889-56905del17bp	Exon 7/ Heterozygous	p.5215fsX223	Novel
				2. g.76430C>T	Exon 14/ Heterozygous	p.R445X	Very recurrent/ Hereditary
			Blood	g.76430C>T	Exon 14/ Mosaic	p.R445X	Very recurrent/ Hereditary
**669**	Bilateral	Neonatal/Enucleation/ Chemotherapy	Blood	g.ENOX1-6?_PCDH8-2?del	*RB1*,Centrom&Tel genes deletion	Chromosome 13q14 del	Low frequency/ Hereditary
**670**	Bilateral	21/Enucleation/Chemotherapy/Radiotherapy	Blood	g.64348C>T	Exon 10C>T	p.R320X	Very Recurrent Hereditary
**673**	Unilateral	46/Enucleation	Tumor	1. g.56905-56906delAT	Exon 7: 2bp del	p.L220fsX223	Reported once
				2. g.78250C>T	Exon 17: C>T	p.R556X	Very recurrent/ Nonhereditary
			Blood	Absence of mutation			
**678**	Bilateral	1/Enucleation/ Chemotherapy	Blood	g.76460C>T	Exon 14	p.R455X	Very Recurrent/ Hereditary
**Mother**	Unilateral		Blood	g.76460C>T	Exon 14	p.R455X	Hereditary
**75**	Bilateral	10/Enucleation/Chemotherapy/Radiotherapy	Blood	g.59695C>T	Exon 8	p.R255X	Very Recurrent/ Hereditary
**686**	Unilateral	1/Enucleation	Tumor	1. g.76478insT	Exon 14	p.K462fsX	Novel
				2..*ITM2B-5*-*RB1*Exons1-2 del	Deletion of Exons 1 and 2		
			Blood	g.76478insT	Exon 14	p.K462fsX	Hereditary
**687**	Bilateral	14days/Enucleation/ Chemotherapy	Blood	g.162035delT	Exon 22	p.S755fs3X	Novel/Hereditary
**689**	Unilateral	55/Enucleation	Tumor	1. g.5484dupA	Exon 2	p.P67fs44X	Reported once
				2. LOH			
			Blood	Absence of mutation			Nonhereditary

Mutation description according to den Dunen and Antonarakis nomenclature using the genomic sequence of GenBank (L11910.1); del: deletion; ins: insertion; dup: duplication;Centrom: centromeric; Tel: telomeric The references for the *RB1* gene variants have been reported in the Leiden Open Variation Database for *RB1* gene (http://rb1-lovd.d-lohmann.de).

### *RB1* mutations

The *RB1* mutations are described in Table 1. A total of 15 mutations were identified in a cohort of 34 patients. Twenty five of the patients had unilateral RB and six of them had available tumor samples. Germline mutations were identified in eight out of nine patients with bilateral/trilateral RB (89%) and in five out of 25 sporadic unilateral patients (20%). Somatic mutations were found in the tumor of four out of five unilateral patients with available tumor samples. The identified mutations were distributed throughout the *RB1* gene and included 12 nonsense/frameshift mutations in ten patients (including two mutations in two of the tumors), one splice-site mutation in two patients, one germline deletion of the whole *RB1* gene, a partial somatic deletion in the *RB1* gene, and two missense mutations. In addition, one variant which appears to be a polymorphism, was detected in two unrelated RB patients and in one asymptomatic parent.

#### Nonsense and frame-shift mutations

**Fig 1:**
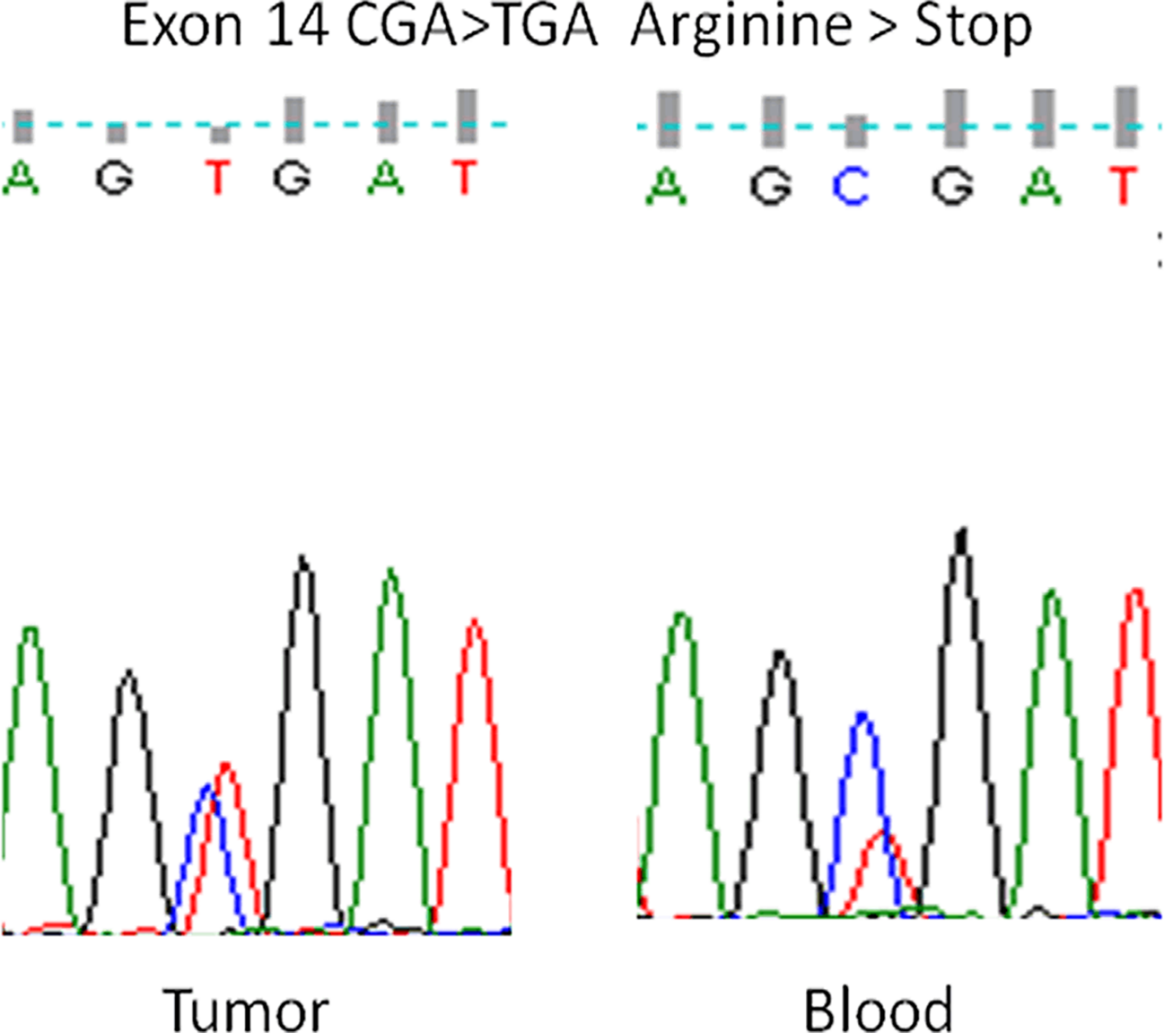
Sequence analysis of exon 14 in a unilateral RB patient (#668). The heterozygous C to T transition generated a stop codon TGA, in which the mutant T peak was lower in height than the wild type C peak in DNA from blood, whereas in DNA from tumor the mutant T and wild type C peaks were similar suggesting a mosaic mutation.

Nonsense germline mutations were identified in one unilateral RB patient (#668) and three bilateral RB patients (#670, #678, #687). The unilateral patient was diagnosed late, at the age of four, which does not correlate with a nonsense germinal mutation. However, in this patient´s blood, the height of the peak of the mutant base (T) was ~40% lower than the height of the peak of the wild type base (C), whereas in the tumor, the heights of the peaks of the mutant and the wild type bases were similar (Fig 1). Thus, the germline mutation may not be present in all of the patient´s cells, such as the leukocytes, suggesting the coexistence of two different cell types, one with a mutant copy of *RB1* and a wild type copy of *RB1* and the other with two wild type copies. This mosaicism could lead to a milder form of RB and could explain the unilateral form and the late tumor onset. It should be noted that this mutation was in the first bp of exon 14, thus, it could also affect exon splicing. The second somatic mutation in this patient was a 17-bp deletion in exon 7, leading to a frame-shift mutation and a premature stop codon. One bilateral patient with a nonsense germline mutatrion (#678) was a familial case, in which the mother had unilateral RB. Two other bilateral patients with nonsense germline mutations (#670, #687) were enucleated and received chemotherapy and radiotherapy. All of the non-sense mutations were the very recurrent C to T transitions in CGA codons in different exons.

Four germline frameshift mutations including one-bp deletions of an A or a T and one-bp insertion of a T were identified in exons 9 and 14 in two unilateral patients (#663, #686), who were diagnosed at early age, in exon 22 of a bilateral patient (#687) and in exon 7 of a trilateral patient (#666). Two of the mutations in patients #686 and #687 were novel, and the other two mutations in patients #663 and #666 have rarely been reported. Three somatic frame-shift mutations were identified: a 17-bp deletion in exon 7 of patient #668, a two-bp deletion in exon 7 of patient #673 and a one-bp duplication in exon 2 of patient #689. In two of the tumors the *RB1* gene was inactivated by two small mutations and in the third tumor *RB1* was inactivated by a chromosomal loss, which is the second most frequent type of mutation.

#### Splice-site mutations

The G to A transition at the conserved donor splice-site of intron 1 was identified in two unrelated bilateral patients (#658 and #660), in patient #658 the mutation was heterozygous in the blood and hemizygous in the tumor because the second mutation in the tumor was a loss of heterozygosity (LOH) mutation. The transition from G to A reduced the splicing score from 96.67 to 69.83, with the proximal cryptic splice-sites at c.137+45G and c.133G (exonic). The use of either of these sites results in frame-shifts and premature generation of stop codons.

#### Large deletion

An entire *RB1* gene deletion, including neighboring centromeric and telomeric genes, was identified by MLPA in the constitutional DNA of bilateral patient #669. This patient was diagnosed at birth and treated with an initial session of intra-arterial chemotherapy, enucleation and an additional session of chemotherapy.

#### Missense mutations

The missense mutations are not as harmful as the nonsense/frameshift mutations because they do not lead to a loss of pRB protein, but rather they reduce pRB function. Therefore, missense mutations originate few tumors (unilateral RB) or no tumors, being the diseased eye ratio (the sum of affected eyes/number of mutation carriers) lower than for nonsense/frameshift mutations (~2), which indicates low penetrance.

**Fig 2:**
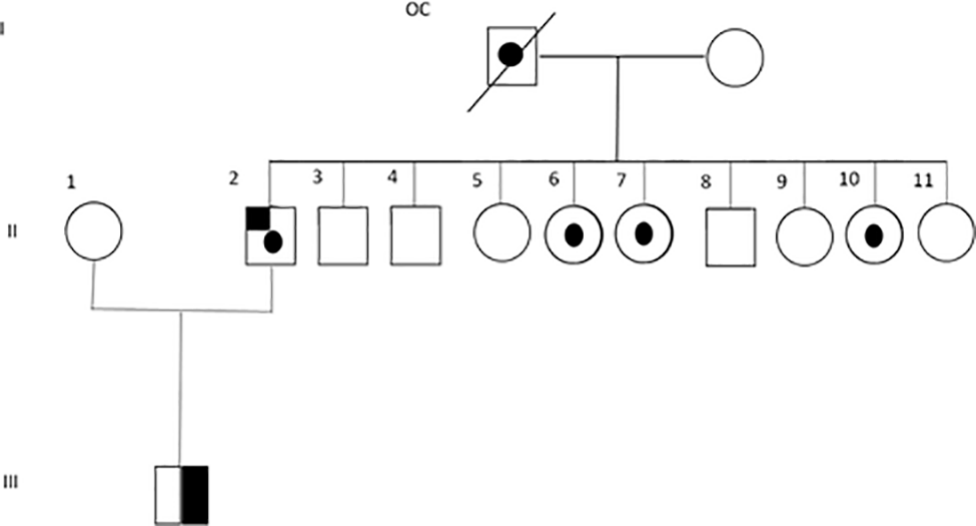
Pedigree of a family with low penetrance retinoblastoma. A unilateral RB patient (#661) carried a germline missense mutation in exon 7 which changed the amino acid leucine by proline (p.Leu223>Pro). The patient inherited this mutation from his father in whom this mutation led to a retinoma. Three out of nine of the father´s siblings also carried the mutation, but they were asymptomatic and none of them had children yet. OC: obligate carrier; half-blackened symbols: unilateral RB; dotted symbols: unaffected carriers; upper-left blackened symbol: retinoma; dashed symbol: deceased.

Two unilateral patients (#661 and #665) carried germline missense mutations inherited from their asymptomatic parents. However, one of these parents the father of patient #661 was found to carry a benign retinoma tumor, thus the same mutation originated retinoma in the father and retinoblastoma in his son [9]. This family is an example of low penetrance since three asymptomatic paternal siblings also carried the mutation, being the diseased eye ratio 0.2 (1/5) (Fig 2). The other unilateral patient (#665) inherited the mutation from his asymptomatic mother.

#### In-frame insertion

**Fig 3:**
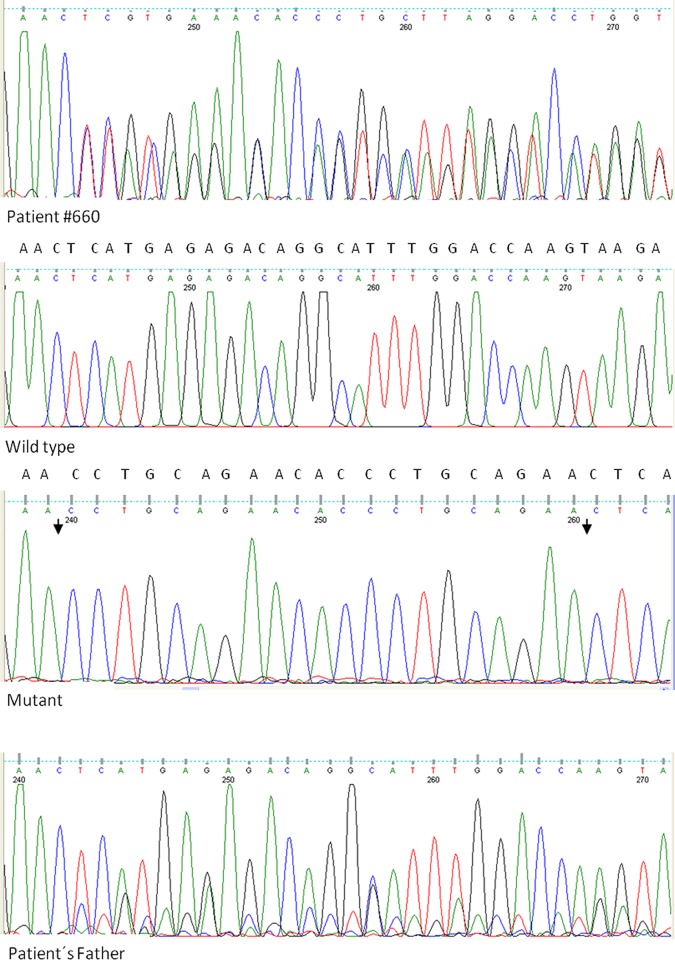
Sequence analysis of a 21-bp heterozygous insertion in exon 20 of *RB1*from a bilateral RB patient (#660). Both, the mutant and wild type copies of exon 20 were retrieved by cloning. The site of insertion in the mutant copy is indicated between the arrows.

A 21bp insertion in exon 20 was identified in the constitutional DNA of a bilateral patient (#660) which was validated by cloning the PCR product into the pGEM-T vector. Five recombinant clones were analyzed, three of them contained the mutant form of exon 20 and the other two contained the wild type form of exon 20, thus confirming a heterozygous insertion (Fig 3). The inserted sequence was a nine-bp (CCTGCAGAA) direct repeat which encoded the amino acids proline (P), alanine (A) and glutamic acid (E) in tandem and these tandem repeats were separated by a histidine codon (CAC). The seven amino acid insertion (PAEHPAE) located in the pocket B domain (p693) of pRB. Although this domain is an ordered structure the RbPL and RbC domains that flank the pocket are flexible and may allow for different conformations, such as that in which the seven amino acids are in an external chain.

The same 21-bp heterozygous insertion was present in the asymptomatic father of the patient but at a lower level (the height of most of the mutant peaks was ~40% lower than the height of the wild type peaks) suggesting mosaicism. In addition, the same 21-bp insertion was found in another unrelated RB patient (#663) and was also found at a lower level than the wild type sequence (30%). Both patients (#660 & #663) carried an additional mutation in their constitutional DNA: a splice-site mutation (#660) and one-bp deletion (#663). The presence of these additional disease-causing mutations confirms the non-pathogenic nature of the 21bp insertion, which has not been reported in the Leiden Open Variation Database for the *RB1* gene (http://rb1-lovd.d-lohmann.de).

## Discussion

The percentage of unilateral, bilateral, trilateral, and familial RB cases in the cohort of patients studied up to date was 53%, 36%, 2%, and 9% respectively. The mean age at diagnosis was 24 months for unilateral patients, 12 months for bilateral patients and two months for trilateral patients. Three of the four trilateral patients in our cohort died at seven months, two years and four years of age. The fourth trilateral patient survives and is 7 years old.

The sensitivity of the methodology used to identify the *RB1* mutations in the blood of bilateral/trilateral patients and in the tumors of unilateral patients was approximately 90%. The nonsense/frame-shift and /splice-site mutations and the large deletion accounted for 83% of the mutations and associated mostly with severe phenotypes except in two unilateral patients. One of these unilateral patients was diagnosed at an early age (nine months) thus bilateralization may occur in the future, and the other unilateral patient diagnosed at a late age probably has a mosaic mutation.

Mutations in the donor splice-site in intron 1 have important consequences. In addition to altering the splicing of exons they affect the initiation of transcription. It has been shown that introns influence the early steps of transcription. The 5´donor site stimulates the pre-initiation complex formation via the U1snRNA and the recruitment of transcription initiation factors [27]. Thus, alterations in the promoter proximal splice-site lead to a significant reduction in nascent transcription [28]. Although this splice-site mutation is rare and has only been reported three times in http://rb1-lovd.d-lohmann.de), we identified it in two bilateral RB patients.

The deletion of the entire *RB1* gene and the neighboring centromeric and telomeric genes in a bilateral patient indicated a large genetic loss, which was confirmed by cytogenetic analysis (13q12.3–14.3 interstitial deletion). This type of *RB1* deletions was found primarily in the unilateral patients of our cohort, which agrees with the hypothesis of DiCiommo et al. [29], who stated that the genes that neighbor *RB1* may be vital for the cell and if their deletion is followed by a second LOH *RB1* mutation, the cell could not survive, thus the tumor transformation would occur in only a few cells. The development of bilateral tumors as a consequence of gross rearrangements could be explained by the presence of a second point mutation that inactivates *RB1* in the retinoblasts, without the loss of neighboring genes.

Low penetrance mutations such as missense mutations, associated with milder phenotypes, including unilateral tumors. Two unilateral patients who carried missense mutations, inherited from their asymptomatic parents, were diagnosed at late ages. Both of these patients´ mutations occurred at highly conserved (across species) amino acid residues in the amino terminal domain of pRB. One of the mutations was in exon 7 (pRB223L>P) and the other mutation was in exon 1 (pRB20P>L, within the repeat of several P residues). The amino terminal region of pRB is a structured domain that maintains the proper conformation of the pocket domain for the binding of E2F transcription factors [15]. However, it has been suggested that mutations in the amino-terminal domain frequently result in low penetrance RB [30]. Both of the missense mutations identified in this study occurred in repetitive nucleotide sequences. One of the mutations was within the nucleotide sequence CCTT, and was a T to C transition in exon 7. The other mutation was in a region containing repeated C´s and was a C to T transition in exon 1. The frequency of these mutations was found to be much higher than expected [31]. Low penetrance mutations may have a subtle effect on the tertiary structure of pRB such as that pRB retains residual function. This type of mutation is known as a weak allele, and this variant of pRB can suppress tumorigenesis in the biallelic state but not in the monoallelic state [19].

A polymorphic variant, the 21bp in-frame insertion in exon 20 of patient #660 resulted in an apparently non-deleterious change. However, it was difficult to assess the alterations that occurred in the pRB protein upon insertion of the seven amino acids. Several possible conformation structures could have resulted, but the most likely, according to Procheck was an external chain of seven amino acids. (Ramachandran plot: 90.2% core 7.4% allow 1.7% gener .6% disall). This conformation would likely result in insignificant changes in the pRB structure.

The data obtained in this study are crucial for genetic counseling and for further understanding the biology of retinoblastoma. The mutations identified are useful for the development of treatments that suppress nonsense mutations and for development of other RB gene therapies. Another utility of the knowledge of an individual's RB-causing mutation can be used during pre-implantation analysis to select embryos without that mutation.

## Conclusions

In this study we identified five novel *RB1* mutations. Two rare *RB1* mutations associated with bilateral RB and included the donor splice-site mutation and the large deletion of *RB1* gene along with several centromeric and telomeric genes. Furthermore, germinal mutations were identified in 20% of the unilateral patients, they included low penetrance and mosaic null mutations.

Rare clinical presentations of RB, such as trilateral tumors, RB associated with schwannoma and a rare benign tumor retinoma were identified among the patient cohort. Further identification of somatic mutations in two unilateral patients was useful to rule out hereditary predisposition. These results are relevant to provide genetic counseling to the affected families.
